# Stem Cells in Pituitary Tumors: Experimental Evidence Supporting Their Existence and Their Role in Tumor Clinical Behavior

**DOI:** 10.3389/fendo.2019.00745

**Published:** 2019-10-25

**Authors:** Giovanna Mantovani, Elena Giardino, Donatella Treppiedi, Rosa Catalano, Federica Mangili, Anna Spada, Maura Arosio, Erika Peverelli

**Affiliations:** ^1^Department of Clinical Sciences and Community Health, University of Milan, Milan, Italy; ^2^Endocrinology Unit, Fondazione IRCCS Ca' Granda Ospedale Maggiore Policlinico, Milan, Italy; ^3^PhD Program in Endocrinological Sciences, Sapienza University of Rome, Rome, Italy

**Keywords:** pituitary tumors, stem cells, tumourigenesis, resistance, invasiveness, recurrence

## Abstract

Although generally benign, pituitary tumors frequently show local invasiveness and resistance to pharmacological therapy. After the demonstration of the existence of pituitary gland stem cells, over the past decade, the presence of a stem cell subpopulation in pituitary tumors has been investigated, analogous to the cancer stem cell model developed for malignant tumors. This review recapitulates the experimental evidence supporting the existence of a population of stem-like cells in pituitary tumors, focusing on their potential role in tumor initiation, progression, recurrence and resistance to pharmacological therapy.

## Introduction

Pituitary tumors represent 10–25% of intracranial neoplasms. They can be classified based on their secretory activity into non-functioning pituitary tumors (NFPTs) or hormone-secreting tumors, including prolactin (PRL)-, growth hormone (GH)-, and adenocorticotropic hormone (ACTH)-secreting tumors. Pituitary tumors may cause visual field deficits and neurologic manifestations from mass effects and/or endocrine syndromes with specific signs and symptoms.

The pathogenetic mechanisms of pituitary tumourigenesis may be genetic or epigenetic and include cell cycle dysregulation, activation of oncoproteins, alterations in growth factors signaling, changes in the intrapituitary microenvironment, and germline or somatic mutations [reviewed in (1)]. In rare cases, germline pathogenetic mutations predispose individuals to pituitary tumourigenesis, often in the setting of familial genetic syndromes, whereas in the majority of sporadic cases, the exact molecular pathogenetic mechanism remains unknown.

Neurosurgery by the transsphenoidal approach is the treatment of choice in the majority of cases, except for PRL-secreting tumors, for which dopamine receptor type 2 (DRD2) agonists (DAs) represent the first-line therapy. Pharmacological treatment of GH- and ACTH-secreting tumors is based on the use of somatostatin (SS) analogs (SSAs). However, a subset of patients (approximately 10% of PRL-, 30% of GH-, and 50–70% of ACTH-secreting tumors) is resistant to these drugs (2–5).

Although classified as benign, pituitary tumors are frequently highly invasive into surrounding tissues (6) and can exhibit clinically aggressive behavior. Invasive tumors are associated with incomplete tumor resection and a high rate of recurrence (7, 8). Pituitary carcinomas are extremely rare, accounting for only 0.1–0.2% of pituitary tumors, but the prognosis is very poor, and 66% of patients die within the first year (9).

The discovery of a population of stem-like cells in pituitary tumors has raised the question about the role these cells in tumourigenesis, growth, invasiveness, recurrence and resistance to pharmacological treatment, similar to the cancer stem cell (CSC) model theorized for malignant tumors.

## Cancer Stem Cells: Characteristics, Markers, and Therapies

The cancer stem cell model states that tumor initiation, progression, growth and recurrence are promoted by a subpopulation of tumor stem cells (10). CSCs were first identified and studied in human leukemia (11, 12) and then in different types of solid tumors, including breast (13), brain (14) and liver (15) cancers. Although most studies have demonstrated that CSC are relatively rare in a number of tumor types, recent works show that tumorigenic potential is a common attribute of melanoma cells (16, 17).

CSCs have indefinite self-renewal capacity, an ability shared with normal tissue stem cells. However, unlike normal stem cells, CSCs also have tumor-generating potential (10). Moreover, CSCs have multilineage differentiation potential and are able to give rise to progenitors that can differentiate into all cell types that compose the bulk of the tumor mass. The multipotency of CSCs provides an explanation for intra-tumor heterogeneity (17). However, committed cells and even completely differentiated cells retain the ability to dedifferentiate, suggesting a high level of plasticity instead of a hierarchical one-direction differentiation (17). In this scenario, a CSC is considered a cell state that can switch from stem cell to differentiated cell, and vice-versa.

Based on the CSC model, while CSCs trigger tumor recurrence and metastatic spreading, the non-CSC population of the tumor does not contribute to the long-term growth of the tumor, as it can undergo only transient proliferation. In addition, CSCs are resistant to chemotherapy and radiotherapy (18) due to their specific features, e.g., slower rate of proliferation or quiescence state, small size, high expression of drug efflux pumps, alterations in apoptosis pathways and expression of telomerase.

The origin of CSCs is still uncertain. It has been proposed that they derive from the oncogenic transformation of normal tissue-specific stem cells (19, 20), progenitors or differentiated cells after reprogramming and dedifferentiation (21).

Signaling pathways that regulate the balance between self-renewal and differentiation of both normal and cancer stem cells are classically associated with oncogenesis, such as Notch, Sonic hedgehog, octamer-binding transcription factor 4 (OCT4) and Wnt. For example, overactivation of the Wnt/β-catenin pathway leads to the transformation of intestinal crypt stem cells into CSCs (19).

Both normal stem cells and CSCs reside in specific microenvironments called niches that are mainly composed of fibroblasts and endothelial, mesenchymal and immune cells, are crucial for the regulation of their self-renewal, activation and differentiation (22) and contribute to drug resistance of the tumor (23).

CSC surface markers have not been clearly established, mainly because of significant inter- and intra-tumor heterogeneity. CSCs isolated from different cancers share markers typical of physiological stem cells, such as CD24, CD44, CD90, CD123, CD133, OCT4, SOX2, Nanog, Nestin, c-kit, ABCG2, and ALDH1 (17, 24). In many cases CSCs can only be identified by a combination of different markers, and most of the CSCs markers are also expressed by normal stem cells (17, 25).

CSCs have the ability to form multicellular three-dimensional floating spheres *in vitro* when grown in non-adherent serum-free conditions (26, 27). This *in vitro* assay allows the growth and propagation of CSCs, and their subsequent molecular characterization.

The gold standard assay for identifying CSCs is serial tumor transplantation at limited dilutions in immunodeficient mice, which can assess both self-renewal and multipotency of a putative CSC subpopulation (26). Caveats reside in the choice of suitable CSC surface markers for the isolation of CSCs for transplantation and technical issues such as the dissociation of the tumor mass, which inevitably results in the disruption of cell-to-cell contacts, attachment to the extracellular matrix, and signals from the microenvironment, with possible consequences on tumor-initiating potential.

Recently developed experiments of genetic lineage tracing and cell ablation have overcome some of these limitations and confirmed *in situ* that many solid tumors contain stem cells in dedicated niches (20, 28–30).

According to the CSC model, complete tumor eradication requires a combination of conventional treatment directed toward bulk tumor cells with CSC-targeted drugs to prevent recurrence, resistance and metastasis, all of which are sustained by the CSC population. The first idea of anti-CSC therapy was based on early observation that leukaemic cells were blocked in an undifferentiated state. Drugs able to induce terminal differentiation of CSCs were thus proposed (differentiation therapy), with successful applications in patients with leukemia (31). However, limited evidence of differentiation therapy efficacy is available in solid tumors [revised in (32)]. A recent study in osteosarcoma stem cells demonstrated that the ROCK inhibitor fasudil significantly suppressed cell growth *in vitro* and tumourigenicity *in vivo* by inducing cell differentiation (33). In cultured CSCs of non-small cell lung cancer, an inhibitor of GSK3β exhibited a strong antiproliferative effect (34). In glioblastoma and neuroblastoma the inhibition of AKT/mTOR pathway selectively targeted the CSC population (35).

Another strategy is based on antibodies targeting CSCs, but the main limitation of this approach resides in the identification of reliable CSC-associated antigens and in possible damage to normal stem cells. The use of markers differentially expressed on normal stem cells and CSCs has allowed the specific targeting of leukemia stem cells in acute myeloid leukemia (36). An attractive alternative to directly targeting CSCs is represented by targeting their niche, e.g., by blocking stem cell niche signals (10).

## Tumor Stem Cells (TSCs) in Pituitary Tumors

The CSC theory was initially developed for malignant tumors in which CSCs were originally isolated and characterized. However, the identification of normal stem cells in the adult pituitary gland [revised in (37)] has prompted the investigation of the presence of a CSC subpopulation in benign pituitary tumors. In the last decade, experimental evidence has accumulated demonstrating that it is possible to isolate cells from human pituitary tumors that fulfill some or all the features typical of CSCs, namely, clonogenic ability *in vitro*, expression of stem cell markers, ability to grow as rounded cell spheres, multipotency, resistance to chemotherapeutic agents, high efflux capacity, and propagation of tumor tissue following xenotransplantation (Tables 1, 2). Since pituitary tumors are mostly benign, these cells are more correctly referred to as ‘tumor stem cells' (TSCs) (44).

**Table 1:** Published studies describing isolation of TSCs from human pituitary tumors.

**Study**	**Ability to grow as cell spheres**	**Expression of stem cells markers**	**Expression of pituitary specific markers**	**Ability to self-renew**	**Differentiation in pituitary hormone-producing cells**	**Resistance to chemotherapeutics**	**Resistance to DAs/SSAs**	**High efflux capacity**	***In vivo*** **tumourigenicity**	
									**Mouse**	**Zebrafish**
Xu et al. (38)	Yes	Yes	Yes	Yes	Yes	Yes	ND	ND	Yes	ND
Chen et al. (39)	Yes	Yes	ND	Yes	ND	ND	ND	ND	Yes	ND
Mertens et al. (40)	Yes	Yes	ND	Yes	Yes	ND	ND	Yes	NO	ND
Manoranjan et al. (41)	Yes	Yes	ND	Yes	ND	ND	ND	ND	Yes	ND
Würth et al. (42)	Yes	Yes	ND	Yes	Yes	ND	NO	ND	NO	Yes
Peverelli et al. (43)	Yes	Yes	Yes	Yes	ND	ND	NO	ND	ND	Yes

*The assessment of the main features typical of CSCs is reported. ND, Not Determined*.

**Table 2:** Stem cell markers expressed in TSCs spheres isolated from human pituitary tumors.

	**Xu et al. (38)**	**Chen et al. (39)**	**Mertens et al. (40)**	**Manoranjan et al. (41)**	**Würth et al. (42)**	**Peverelli et al. (43)**
CD133	X	X			X	
CD15				X		
CD90	X					X
CXCR4					X	
DLL1	X					
JAG2	X					
KLF4						X
MSI	X					
NANOG					X	
Nestin	X	X	X		X	X
NOTCH1					X	
NOTCH4	X					
OCT4	X				X	X
SOX2			X		X	X

### Isolation of TSCs From Pituitary Tumors

**Figure 1:**
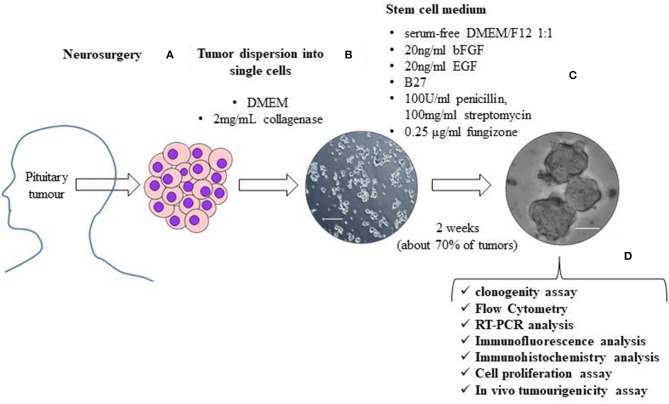
The figure shows a schematic representation of the isolation and characterization of TSCs from surgically removed pituitary tumors. Human pituitary tumors tissues, obtained by the transsphenoidal route, are mechanically and enzymatically (with 2 mg/mL collagenase in DMEM) dissociated **(A)**. To obtain TSCs spheres, tumor cells are resuspended in stem cell permissive medium (serum-free DMEM/F12 1:1 medium) in the presence of 20 ng/ml basic fibroblast growth factor (bFGF), 20 ng/ml epidermal growth factor (EGF), B27, 100 units/ml penicillin, 100 mg/ml streptomycin and 0.25 μg/ml fungizone **(B)**. About 2 weeks after cell seeding in stem cell permissive medium, non-adherent round cell spheres appear in a subset (about 70%) of cultured tumor cells **(C)**. Spheres are then phenotypically characterized for self-renewal ability, stem-cell markers expression, biological behavior and *in vivo* tumourigenicity **(D)**. Scale bar 50μm.

The first study describing a population of cells in pituitary tumors presenting CSC properties was published in 2009 (38). These cells, isolated from one NFPT and one GH-secreting tumor, were able to form floating spheres when grown in stem cell-permissive medium (DMEM/F12, B27 supplement, bFGF, and EGF) (Figure 1), as described for normal pituitary stem cells (45); expressed stem cell markers (OCT4, CD133 NOTCH4 and Nestin) (Table 2) and pituitary-specific markers (PIT1 and <GSU); and could be subcloned in culture (self-renewal assays) and differentiated into pituitary hormone-producing cells. In addition, these TSCs displayed resistance to chemotherapeutics (carboplatin and etoposide), that might be due to the upregulation of anti-apoptotic genes (38) or to an upregulation of multidrug transporters ABCB1 and ABCG2 (40).

These TSCs were able, when transplanted into the forebrains of immunodeficient NOD/SCID mice, to give rise to tumors that recapitulated the phenotypes of human primary tumors, although convincing evidence was not provided. This study presents some inconsistencies regarding hormone production by TSCs derived from NFPT that can produce LH and by TSCs derived from GH-secreting tumors that can secrete PRL and TSH after stimulation. These observations suggest that spheres may include differentiated, hormone-producing cells derived from the differentiation of stem cells, even if cultured in stem cell-permissive medium.

The hypothesis of the existence of pituitary TSCs was further supported 5 years later by a study by Chen et al. (39) that isolated pituitary tumor cells that were grown as floating spheres *in vitro*, expressed the neural stem/progenitor cell markers CD133 and Nestin, and were able to generate daughter cells that could differentiate into three neural lineages (Tables 1, 2). These cells generated slow-growing, synaptophysin-positive tumors after subcutaneous xenotransplantation into SCID mice. The limitations of this study reside in the choice of markers, whose specificity is controversial, and in the lack of demonstration of production of pituitary hormones.

One year later, Mertens and colleagues found a side population (SP) composed of cells with high efflux capacity (40) in pituitary tumors, as previously described for normal pituitary stem cells (46). The SP, when purified from endothelial and immune cells, expressed a panel of CSC markers, including CD44, CXCR4, KIT, KLF4, SOX2, and Nestin, overexpressed genes related to epithelial-to-mesenchymal transition (EMT) and angiogenesis, formed floating spheres in culture, had clonogenic potential and differentiated into pituitary hormone-producing cells. However, in contrast with previous studies, these cells were not able to originate tumors following xenotransplantation into NOD-SCID mice (40) (Tables 1, 2). Tumor-forming potential in mice was instead exhibited by an SP with high efflux capacity that was identified in the mouse pituitary corticotrope tumor cell line AtT20 (40) and by a subpopulation of murine stem-like cells that was isolated from pituitary tumors spontaneously occurring in Rb± mice (47).

A subsequent study (41) on differential gene expression profiles and flow cytometric characterization identified CD15, a CSC marker in other brain tumors, as a marker for pituitary TSCs. The CD15^+^ cell subpopulation displayed high sphere-forming capacity, elevated SOX2 gene expression and tumourigenic potential in mice.

In contrast, the lack of tumor-generating ability of human pituitary TSCs in mice was reported in another study, in which stem-like cells were successfully isolated from 38 of 56 pituitary tumors of different types (GH-, GH/PRL-, and ACTH-secreting tumors and NFPTs) (42).

Since contrasting data have been reported about the *in vivo* tumourigenicity in mice (Table 1), it can be hypothesized that the failure of this assay is due to the typical clinical behavior of pituitary tumors, which is characterized by slow growth, as well as to methodological caveats related to the use of *in vitro* cultures of stem cells prior to transplantation or to the isolation of stem cell populations with different proliferative rates. Moreover, we can speculate that benign tumors may depend more on their niche than malignant tumors, and thus the lack of a proper microenvironment in mice can explain the failure of tumor formation after xenograft (48). In addition, the *in vivo* results of the available studies remain questionable and not entirely convincing. The major issues reside in the excessive number of transplanted cells, lack of data on long-term growth and serial *in vivo* transplantation.

On the other hand, the pro-angiogenic and invasive potential of pituitary TSCs was successfully demonstrated in zebrafish embryos (42, 43) (Table 1), an alternative model that allows the detection of *in vivo* biological behaviors of tumor cells in a shorter time than that in mice, which is more compatible with the slow growth rate of pituitary tumors.

Würth et al. (42) and Peverelli et al. (43) isolated TSCs growing as floating spheres from a high number of NFPTs (*n* = 32) with a success rate of sphere formation nearly identical to that which was previously reported. In both of these studies, the sphere-forming cells were able to self-renew in culture and expressed several markers of stemness, including pluripotent embryonic stem cell markers (SOX2, OCT4, KLF4, and Nestin) (Table 2). In addition, NFPT-derived TSCs expressed embryonic pituitary-specific transcription factors involved in gonadotroph differentiation (DAX1, SF1, and EGR1), which is consistent with the gonadotroph origin of most NFPTs (43), and mature hormones (38, 40). Interestingly, the expression of pituitary tumor-specific receptors, SS receptors type 2 (SSTR2) and 5 (SSTR5) or DRD2 has been detected in cells in the spheres (42, 43). These observations suggest the presence of committed precursors in spheres cultured *in vitro* in stem cell-permissive medium.

Pituitary TSCs demonstrate long-term proliferation ability of up to 1–2 months in culture (42, 43), in striking contrast with differentiated cells from the same tumors, which actively proliferate only for 1 week (43), and with pituitary cell primary cultures, which usually survive *in vitro* for only a few days.

The analysis of stem cell marker expression in NFPT tissues revealed the expression of SOX2, OCT4, KLF4, and EGR1 (43). Interestingly, SOX2-positive cells, sparsely distributed in the tissue sections and representing approximately 4% of pituitary cells of NFPT tissues, were negative for pituitary hormones, and only 1% of SOX2^+^ cells co-expressed LH (43). Similarly, in GH-secreting tumor tissues, no co-expression of GH with SOX2, OCT4, or Nestin was observed (42). Overall, these data indicate the presence of a hormone-negative cell subpopulation expressing stem cell markers in pituitary tumors.

### Role of Pituitary TSCs in Pituitary Tumor Resistance to Medical Therapy

The molecular basis of pituitary tumor resistance to pharmacological therapy with DAs and SSAs is still poorly understood. The possible involvement of a drug-resistant TSC population has been independently investigated by two different groups with similar findings (Table 3).

**Table 3:** The table summarizes the results of the published studies supporting or not a possible role of pituitary TSCs in human pituitary tumors clinical behavior.

	**Role of TSCs**	
	**Yes**	**No**
Resistance to medical therapy	(38) (carboplatin, etoposide)	(43) (BIM53097, BIM23120); (42) (BIM-23A760)
Invasiveness	(39, 40, 42, 43)	–
Recurrence	(41)	(49)

Peverelli et al. (43) tested the antiproliferative effects of the specific DRD2 agonist BIM53097 and SSTR2 agonist BIM23120 on cultured cells derived from NFPT tissues immediately after dispersion prior to sphere formation and on TSC spheres. Cell proliferation and cell cycle progression were evaluated by measuring BrdU incorporation, cyclin D3 and cyclin-dependent kinase inhibitor p27 levels. They found that in the subset of NFPTs in which DRD2 or SSTR2 agonists inhibited bulk tumor cell proliferation, the antiproliferative effect was maintained in the corresponding spheres.

In agreement, Würth et al. (42) observed a reduction in TSC survival measured by MTT assay upon somatostatin/dopamine chimera BIM-23A760 incubation.

Overall, these results suggest that pituitary TSCs are not characterized by resistance to the drugs currently used in the treatment of pituitary tumors, ruling out the hypothesis that these cells are responsible for the pharmacological resistance observed in a subset of patients, as also supported by the lack of difference in the frequency of TSC sphere formation between NFPTs that were resistant or sensitive to these drugs *in vitro* (43).

On the other hand, based on these data, pituitary TSCs can be considered a good target for pharmacological approaches using DAs and SSAs, supporting the use of these agents in adjuvant medical therapy. In agreement, it has been reported that DA therapy in patients with NFPTs was associated with a decreased prevalence of residual tumor regrowth after surgical resection (50).

Interestingly, TSC spheres derived from resistant NFPTs were significantly larger than those obtained from sensitive ones (43), suggesting an increased proliferative activity of TSCs in resistant tumors, which is consistent with the notion that resistance is associated with increased pituitary tumor aggressiveness.

### Role of Pituitary TSCs in Pituitary Tumor Invasiveness

Since only approximately 70% of pituitary tumors gave rise to TSC spheres in culture (42, 43), a possible correlation between this ability and clinical tumor behavior has been hypothesized (Table 3). Comparison of clinicopathological and radiological features revealed that the formation of spheres was positively associated with cavernous sinus invasion, whereas no correlation with sex, age, tumor size, Ki67 and extrasellar extension was found (43). Accordingly, the number of CD133^+^ cells was significantly increased in invasive tumors compared that of non-invasive pituitary tumors, as determined by both immunocytochemistry and flow cytometry assays (39). In this respect, it is of interest to note that the SP isolated from pituitary tumors by Mertens et al. (40) overexpressed gene clusters involved in cell motility and migration. In addition, pituitary TSCs xenografted into zebrafish embryos migrated from the site of injection and showed strong invasive behavior (42, 43).

Together, these data suggest that pituitary TSCs may contribute to the local invasiveness of pituitary tumors.

Further studies are required to evaluate the differential cell migration and invasion ability of TSCs in comparison to that of differentiated bulk tumor cells. Moreover, since it has been shown that DAs and SSAs exert inhibitory effects on the migration and invasion of pituitary tumor cells (51, 52), it would be of great interest to test whether these effects are maintained in TSCs, possibly supporting the use of these agents in the control and prevention of tumor local invasiveness.

### Role of Pituitary TSCs in Pituitary Tumor Recurrence

There is little data available about a possible contribution of pituitary TSCs to tumor recurrence, according to the CSC model (Table 3).

Manoranjan and colleagues found an enrichment of CD15 expression in recurrent tumors compared to that of their matched primary tumors (41), suggesting that CD15^+^ TSCs may promote tumor relapse.

Yunoue et al. (49) reported no correlation between the expression of CD133 in pituitary tumors and the postoperative recurrence rate. Although CD133 is considered a common marker of CSCs in different tumors and its presence has been detected in pituitary tumors (39, 53), it is worth noting that contrasting data have been reported on CD133 expression in TSC spheres derived from pituitary tumors, ranging from positive to negative or low expression (38, 39, 41–43, 54), highlighting the need for the identification of more precise markers for pituitary TSCs.

### Role of Pituitary TSCs in Tumourigenesis and Tumor Progression

Long-term proliferation ability and tumourigenic potential in animal models exhibited by pituitary TSCs suggest that TSCs may play a major role in the initiation process of pituitary tumourigenesis, as well as in pituitary tumor growth, but this hypothesis has yet to be proven.

Recently, non-secreting and aggressive pituitary tumors have been found in mice bearing deletion of the LATS gene, a kinase belonging to the Hippo pathway (LATS/YAP/TAZ signaling), which is crucial in the maintenance of active pituitary stem cell state and in the inhibition of differentiation (55). These murine tumors were composed predominantly of SOX2^+^ cells, suggesting that loss of LATS, obtained by genetic manipulation, drives deregulation of SOX2^+^ pituitary stem cells, leading to tumor formation. Interestingly, overexpression of Hippo pathway components has been reported in the SP of pituitary tumors (40).

In another pituitary tumor type, adamantinomatous craniopharyngioma (ACP), strong evidence supports a paracrine role of TSCs in tumor development and growth by stimulation of the proliferation of adjacent cells. Genetic linage tracing experiments in a mouse model of ACP showed that SOX2^+^ cells targeted with mutant β-catenin did not autonomously give rise to the tumor mass but instead generated the tumor from neighboring cells in a paracrine manner (44, 56, 57).

## Conclusions

In the past decade, the existence of a population of stem cells in pituitary tumors has been established, but the role of these pituitary TSCs in tumor initiation, progression, recurrence and resistance to pharmacological therapy, analogous to the CSC model, needs to be further elucidated. Although pituitary TSCs are not responsible for resistance to DAs and SSAs, their presence seems to be associated with tumor invasiveness and possibly recurrence.

The presence of stem cells in benign pituitary tumors may suggest that CSCs are not associated with malignancy. However, studies on CSCs in pituitary carcinomas or atypical pituitary adenomas would be required to clarify their role in pituitary tumor aggressiveness.

Admittedly, most of the published studies investigated TSCs in NFPTs, whereas little information is available for the other types of pituitary tumors.

Further studies aimed to clarify the relationship between tumor-initiating cells and pituitary TSCs are needed. Once the pituitary TSCs and the mechanisms involved in their regulation have been fully characterized, new therapeutic strategies targeting TSCs and new biomarkers predicting pituitary tumor clinical behavior could be developed.

## Author Contributions

GM, EG, DT, RC, FM, and EP wrote the manuscript. EP, AS, and MA contributed to manuscript revision. All authors read and approved the submitted version.

### Conflict of Interest

The authors declare that the research was conducted in the absence of any commercial or financial relationships that could be construed as a potential conflict of interest.
